# Intradermal Delivery of Dendritic Cell-Targeting Chimeric mAbs Genetically Fused to Type 2 Dengue Virus Nonstructural Protein 1

**DOI:** 10.3390/vaccines8040565

**Published:** 2020-10-01

**Authors:** Lennon Ramos Pereira, Elaine Cristina Matos Vicentin, Sara Araujo Pereira, Denicar Lina Nascimento Fabris Maeda, Rúbens Prince dos Santos Alves, Robert Andreata-Santos, Francielle Tramontini Gomes de Sousa, Marcio Massao Yamamoto, Maria Fernanda Castro-Amarante, Marianna Teixeira de Pinho Favaro, Camila Malta Romano, Ester Cerdeira Sabino, Silvia Beatriz Boscardin, Luís Carlos de Souza Ferreira

**Affiliations:** 1 Department of Microbiology, Institute of Biomedical Sciences, University of Sao Paulo, Sao Paulo 05508-000, Brazil; lennon_rp@hotmail.com (L.R.P.); araujopereirasara@gmail.com (S.A.P.); denicarlina@usp.br (D.L.N.F.M.); rubens.bmc@gmail.com (R.P.d.S.A.); robert_andreata@hotmail.com (R.A.-S.); mfamarante@usp.br (M.F.C.-A.); favaro.mtp@gmail.com (M.T.d.P.F.); 2 Department of Parasitology, Institute of Biomedical Sciences, University of Sao Paulo, Sao Paulo 05508-000, Brazil; elaine.vicentin@gmail.com (E.C.M.V.); masayama@usp.br (M.M.Y.); sbboscardin@usp.br (S.B.B.); 3 Clinical Hospital HCFMUSP, Faculty of Medicine, University of Sao Paulo, Sao Paulo 05403-000, Brazil; francielletg@gmail.com (F.T.G.d.S.); camismalta@gmail.com (C.M.R.); sabinoec@gmail.com (E.C.S.)

**Keywords:** intradermal, DEC205, DCIR2, dendritic cell, NS1 protein, Dengue virus

## Abstract

Targeting dendritic cells (DCs) by means of monoclonal antibodies (mAbs) capable of binding their surface receptors (DEC205 and DCIR2) has previously been shown to enhance the immunogenicity of genetically fused antigens. This approach has been repeatedly demonstrated to enhance the induced immune responses to passenger antigens and thus represents a promising therapeutic and/or prophylactic strategy against different infectious diseases. Additionally, under experimental conditions, chimeric αDEC205 or αDCIR2 mAbs are usually administered via an intraperitoneal (i.p.) route, which is not reproducible in clinical settings. In this study, we characterized the delivery of chimeric αDEC205 or αDCIR2 mAbs via an intradermal (i.d.) route, compared the elicited humoral immune responses, and evaluated the safety of this potential immunization strategy under preclinical conditions. As a model antigen, we used type 2 dengue virus (DENV2) nonstructural protein 1 (NS1). The results show that the administration of chimeric DC-targeting mAbs via the i.d. route induced humoral immune responses to the passenger antigen equivalent or superior to those elicited by i.p. immunization with no toxic effects to the animals. Collectively, these results clearly indicate that i.d. administration of DC-targeting chimeric mAbs presents promising approaches for the development of subunit vaccines, particularly against DENV and other flaviviruses.

## 1. Introduction

Targeting antigens to dendritic cells (DCs) has been repeatedly demonstrated to improve the immunogenicity of subunit vaccines. DCs are specialized in antigen presentation with functions that include the initiation and regulation of host immune responses. An efficient DC-targeting delivery method relies on the genetic fusion of antigens to monoclonal antibodies (mAbs) that specifically bind to endocytic receptors expressed on the surface of DCs [1,2,3]. Among various DC types, CD8α+ and CD8α- subsets have important immunological properties and express the C-type lectin endocytic receptors DEC205/CD205 and DCIR2, respectively [1,3,4]. Studies indicate that CD8α-DCIR2+ DCs are efficient in antigen presentation via major histocompatibility complex (MHC) II molecules and CD4+ T cell activation, while the CD8α+DEC205+ subset performs antigen cross presentation and activation of both CD8+ and CD4+ T cells. On the other hand, both DC subsets are effective in enhancing antigen-specific antibody responses [5,6,7,8]. Despite these promising perspectives, these antigen delivery vaccine approaches have been rarely explored under clinical conditions [1].

Different factors may influence the DC-targeting approach based on the use of chimeric mAbs, including the choice of the DC subset and administration route [1]. Most of the preclinical studies carried out with αDCIR2 or αDEC205 mAbs were performed in mice following delivery via an intraperitoneal (i.p.) route [3,7,9,10,11,12,13]. Moreover, other more accessible administration routes, such as a subcutaneous (s.c.) route, showed equivalent or superior immunological performance [14,15,16,17]. Under clinical conditions, only the DEC205 targeting strategy has been tested, and it was based on the CDX-1401 vaccine, which is composed of a human αDEC-205 mAb fused to the tumor-associated antigen NY-ESO-1. This preparation was safe and immunogenic after either s.c. or intracutaneous administration [18]. Other strategies are being evaluated in clinical trials (NCT01889719 and NCT01834248) through exploration of either the s.c. or intradermal (i.d.) route, but no data are presently available.

The intradermal route has been successfully tested for the delivery of vaccines against different pathogens [19,20,21,22]. For example, a clinical trial of an attenuated dengue virus based on i.d. delivery was shown to be tolerable and induced protective antibody responses [21,23]. Similarly, preclinical studies based on inactivated or subunit DENV vaccines showed that the i.d. delivery route increases the number of DCs at the inoculation site, the generation of neutralizing antibody titers, and CD8+ T cells compared to the effects after other immunization routes [24,25,26,27,28]. Moreover, the i.d. delivery of the Fluzone^®^ influenza vaccine is presently available for human use [29]. Both human and mouse skins demonstrated to have DCs that can be targeted by an αDEC205 mAb, particularly when coadministered with polyinosinic-polycytidylic acid [poly (I:C)] [30,31,32]. Nonetheless, the i.d. administration route has been mainly unexplored for DC-targeting antigen delivery systems despite the presence of large and diverse DC populations in the human and mouse dermis [30,33,34,35].

An important factor in the establishment of a vaccine against dengue virus (DENV) is the need to induce balanced and safe immune responses against all four virus serotypes. This outcome is particularly critical for vaccines based on structural proteins, which can promote antibody-dependent enhancement (ADE) under sub-neutralizing antibody conditions, increasing the risk of severe dengue [36,37,38]. Alternatively, nonstructural proteins are not related to the ADE phenomenon. Dengue virus nonstructural protein 1 (NS1) has been demonstrated to induce protective immunity when presented via different vaccine development platforms, including viral vector, DNA and purified recombinant protein strategies [39,40,41,42,43,44]. NS1 is a conserved glycoprotein among DENV1-4 serotypes that is highly immunogenic and expressed on infected cells, in which it can be associated with the cellular membrane (mNS1) or secreted into the extracellular compartment as a hexamer (sNS1) [42,43,44,45,46]. We have previously demonstrated that DENV2 NS1 targeting to CD8α-DCIR2+ and CD8α+DEC205+ DC populations induced antigen-specific antibodies and T cell-dependent responses following delivery via an i.p. route, which was correlated to enhanced protective immunity against DENV2 [47]. However, the potential of i.d. vaccination using this targeting strategy remains unexplored.

In this study, we evaluated the immunogenicity and safety of DENV NS1 formulations delivered by the i.d. route under preclinical conditions. DENV2 NS1 protein was targeted to DCs using chimeric αDEC-NS1 and αDCIR2-NS1 mAbs. The results indicated that their association through the i.d. delivery route resulted in potent NS1-specific antibody responses, which were accompanied by a modulation of Immunoglobulin G (IgG) subclass profile, increased antigen avidity, and longevity of the induced antibodies. In addition, we demonstrated that none of the strategies tested via the i.d. route induced deleterious side effects, such as platelet malfunctioning or endothelial cell damage.

## 2. Materials and Methods

### 2.1. Ethics Statement

All experiments with mice were conducted according to the Ethical Principles of Animal Experimentation (Federal Law 11.794 (2008)). The protocols were approved by the Institutional Animal Care and Use Committee (CEUA) of the University of Sao Paulo (protocol number 19/2014, approved on 25 February 2014). Male BALB/c mice (6–8 weeks) were bred under specific pathogen-free conditions at the Isogenic Mouse Facility of the Parasitology Department, University of Sao Paulo, Brazil.

### 2.2. Virus and Cell Lines

Dengue 2 virus (DENV2) strain New Guinea C (NGC) (GenBank: AHG97599.1) was used for in vitro cell infection assays. DENV2 propagation was carried out in *Aedes albopictus* clone C6/36 cells cultured in Leibovitz L-15 medium (Vitrocell, Campinas, Brazil) supplemented with 2% fetal bovine serum (FBS) (Life Technologies, Carlsbad, USA). Vero CCL-81 cells were cultured in Minimum Essential Medium Eagle (MEM, Vitrocell, Campinas, Brazil) with 10% FBS. Human umbilical vein endothelial cells (HUVEC) (Lonza, Walkersville, MD, USA) were cultured in Endothelial Basal Medium (EBM^TM^-2, CC-3156, Lonza, Walkersville, MD, USA) supplemented with cell growth Kit (EGM-2 MV, CC-4147, Lonza, Walkersville, MD, USA). Human embryonic kidney cells (HEK-293 cells, CRL-11268, ATCC) were cultured in Dulbecco’s modified Eagle’s medium (DMEM, Life Technologies, Carlsbad, USA) supplemented with 5% ultralow heat inactivated FBS (Life Technologies, Carlsbad, USA), 1× L-glutamine (Life Technologies, Carlsbad, USA) and 1× antibiotic–antimycotic (Life Technologies, Carlsbad, USA).

### 2.3. Expression and Purification of the Recombinant mAbs and DENV2 NS1

The recombinant antibodies were expressed by transient transfection in HEK-293 cells and purified according to a protocol previously described [47]. Briefly, HEK-293 cells were cultured in 150 mm plates (TPP) until reaching 70–80% confluence. After washing (1×) with DMEM without FBS, 20 mL of DMEM (supplemented with 1% Nutridoma-SP (Roche, Mannheim, Germany), 1× L-glutamine and 1× antibiotic/antifungal solution) was added to the cells. For transfection, 10 µg of each plasmid encoding light and heavy chains (previously produced and purified from the recombinant *E. coli* DH5α strain) and 4.5 μg of polyethyleneimine (PEI) (Sigma Aldrich, San Luis, MO, USA) per μg of DNA were diluted in 150 mM NaCl solution. These mixtures were homogenized, incubated for 5 min (RT), and distributed evenly over the plates. Cells supernatant were collected 5–6 days after transfection, clarified at 1000× *g* (30 min), and filtered through 0.22 μm pore filters (Corning, New York, NY, USA). Recombinant antibodies were purified with protein G beads (GE Healthcare, Boston, MA, USA), and their concentrations were estimated by Bradford assay (Pierce, Waltham, USA). Aliquots were stored at −20 °C.

DENV2 NS1 protein was expressed on the recombinant *E. coli* BL21-CodonPlus (DE3)-RIL strain and purified by affinity chromatography after denaturation followed by refolding of the protein, as previously reported [48].

### 2.4. Immunization Regimens

Male BALB/c mice (6–8 weeks old) were inoculated by the i.d. or i.p. routes according to the following immunization groups: αDEC: animals received 2.5 µg of αDEC mAb; αDCIR2: 2.5 µg of αDCIR2 mAb; αDEC-NS1: 2.5 µg of αDEC-NS1 mAb; αDCIR2-NS1: 2.5 µg of αDCIR2-NS1 mAb; rNS1: 1 μg of DENV2 NS1 recombinant protein (NGC strain). All vaccine formulations included 50 µg/animal of poly (I:C) adjuvant, and saline solution was used as the vehicle. Each animal received two doses of the designated vaccine formulation with a 2-week interval between doses. For the immunological memory induction trial, 146 days after the second vaccine dose, the animals were restimulated with NS1 protein at 1 µg/animal. Blood samples from the animals were obtained by submandibular plexus puncture at 14 days after the administration of each dose and centrifuged at 3000 g for 30 min to separate the sera. To monitor the longevity of the humoral response, additional samples were collected on days 45, 90 and 160 of the vaccine protocol. For the evaluation of immunological memory induction, blood samples were also taken 7 and 15 days after restimulation with the administration of NS1 antigen. The obtained samples were stored at −20 °C until use.

### 2.5. ELISA

Flat-bottom 96-well ELISA plates (Corning) were coated with purified DENV2 NS1 (200 ng/well) at room temperature (RT) for 18 h. The plates were washed three times with a phosphate-buffered saline (PBS) solution containing 0.02% Tween-20 (PBS-T). After washing, plates were blocked with 200 µL/well of 5% non-fat milk solution with 1% bovine serum albumin (BSA) in PBS-T for 2 h at RT. After a new wash cycle (3×), 100 µL/well of the serum diluted in diluent solution (5% milk and 0.25% BSA in PBS-T) was added to the wells and incubated at RT for 2 h. After a wash cycle (3×), 50 µL/well of peroxidase-conjugated anti-mouse IgG secondary antibody (Cat.: 115-035-071, Jackson ImmunoResearch Laboratories, Philadelphia, PA, USA) was added to the wells at a 1:2000 dilution in diluent solution. For the determination of IgG subclasses, IgG1 (Cat.: 1070-01), IgG2a (Cat.: 1080-05), IgG2b (Cat.: 1090-01), and IgG3 (Cat.: 1000-05) anti-subclass antibodies (all from Southern Biotech, Birmingham, AL, USA) were added at a 1:2000 dilution. After 2 h of incubation (RT), the plates were washed (3×) and 100 µL of developing solution [10 mg o-phenylenediamine dihydrochloride (OPD) (Sigma Aldrich, San Luis, MO, USA) dissolved in 10 mL of 0.2 M sodium phosphate solution (Synth, Diadema, Brazil) and 0.2 M citric acid (Synth) (pH 4.7) plus 10 μL of H2O2] was added to each well and incubated for 15 min at RT. The reaction was stopped with 50 µL/well of 1 M sulfuric acid. The absorbance at 490 nm (A490) was measured with a plate reader (BioTek, Winooski, VT, USA). Antibody titer values were considered as the dilution value at which the absorbance obtained was at least 0.1 units above of background wells (wells containing no serum).

### 2.6. Antibody Avidity Determination

The antibody affinity index was measured using an ELISA protocol that included an elution step with ammonium thiocyanate, as previously described [49]. Briefly, plates were coated with DENV2 NS1 protein as described in Section 2.5. The serum collected from the immunized mice was tested and normalized at dilutions corresponding to an A490 of 1.0. After incubation with serum, different concentrations of sodium thiocyanate (0–8 M) were added to the wells and incubated for 15 min at RT. After washing, the plates were incubated with Horseradish peroxidase (HRP)-conjugated anti-mouse IgG antibody. The percentage of antibody binding was calculated as A490 in the presence of ammonium thiocyanate × 100 divided by the A490 value in the absence of ammonium thiocyanate. The concentration of ammonium thiocyanate required to disrupt 50% of the antigen-antibody interactions was calculated by linear regression (percentage of bound antibodies versus the corresponding ammonium thiocyanate concentration).

### 2.7. Flow Cytometry Analysis

#### 2.7.1. Recognition of the Native DENV2 NS1 Protein by the Immune Serum Samples

This assay was adapted from a previously described protocol [49]. Briefly, Vero cell monolayers were cultured in 96-well plates (Corning) after seeding (5 × 10^4^ cells/well) with MEM supplemented with 2% FBS (37 °C, 5% CO_2_, 18 h). The cells were infected with DENV2 at a multiplicity of infection (MOI) of 2.0 and incubated for 24 h (37 °C, 5% CO_2_). After infection, cells monolayers were washed 2× with PBS, tripsinized, and fixed/permeabilized with a Cytofix/Cytoperm kit (BD Bioscience) according to the manufacturer’s instructions. Then, the cells were stained with the primary antibodies: anti-E protein 4G2 mAb (ATCC-HB112) (0.5 µg/well), anti-DENV2 NS1 4F6 mAb [50] or serum samples from the immunized mice (1:2000) on ice for 30 min. After washing (2×), the cells were stained (30 min on ice) with the secondary antibody, goat anti-mouse-Alexa Fluor^®^ 488 (Cat.: A11001, Thermo Fisher Scientific, Waltham, USA), after which they were diluted 1:800. Finally, after washing (2×), the cells were suspended in 200 µL of PBS-2% FBS solution and analyzed by an LSR FortessaTM analyzer (BD, Franklin Lakes, NJ, USA). The data obtained were analyzed using FlowJo software (version 10, Tree Star, San Carlo, CA, USA) to determine the percentage of stained Vero cells.

#### 2.7.2. Binding of Mouse Serum to Platelets

Sodium citrate-anticoagulated human whole blood was centrifuged (100× *g* for 20 min at RT) to obtain platelet-rich plasma (PRP). The PRP was centrifuged at 1000× *g* for 10 min at RT, and the supernatant was separated (platelet-poor plasma, PPP). The resulting pellet was suspended in wash buffer (1 mM Ethylenediaminetetraacetic acid [EDTA] in PBS) and centrifuged again (1000× *g* for 10 min at RT). After washing (2×), the platelets were suspended in fixation solution (1% formaldehyde in PBS) and incubated for 10 min (RT). After fixation, the cells were washed (2×) with PBS and counted with a Neubauer camera. Then, a total of 10^6^ cells was incubated (30 min, RT) with sera of immunized animals diluted in PPP (at a final NS1-specific IgG concentration of 2.5 µg/mL) or commercial mouse IgG antibody (Cat.: 010701, Southern Biotech, Birmingham, AL, USA) at the same concentration. After incubation, the cells were washed 2× with PBS and then labeled (30 min, RT) with conjugated goat anti-mouse IgG antibody (Alexa Fluor-488, BD Biosciences, Franklin Lakes, NJ, USA). After washing (2×) with PBS, the samples were analyzed by flow cytometry (FACSCalibur, BD Biosciences). The data obtained were analyzed using FlowJo software (version 10, Tree Star, San Carlo, CA, USA).

### 2.8. Inhibition of Platelet Aggregation

The inhibition of platelet aggregation was determined according to a previously described protocol [51]. PRP and PPP were prepared as described in Section 2.7.2. For the analysis of platelet aggregation, 400 µL of PRP was incubated separately with each immunized animal serum (at a final NS1-specific IgG concentration of 2.5 µg/mL in 400 µL of PRP), commercial mouse IgG antibody (Southern Biotech, Birmingham, AL, USA) or PBS. To measure the platelet aggregation, the samples were analyzed in an aggregometer (490 2D aggregometer, CHRONO-LOG Corporation, Havertown, PA, USA) for 3 min. Then, 20 µM adenosine diphosphate (ADP) was added as an aggregation inducer, and the samples were monitored for an additional 3 min. The aggregometer apparatus was calibrated with PPP set to 0% aggregation. The platelet aggregation rates were calculated as the increase in light transmission over 3 min after the addition of ADP.

### 2.9. Transendothelial Electrical Resistance (TEER) Assay

HUVECs seeded in Transwell^®^ inserts (Corning, Cat.: 3392, 0.143 cm ^2^ growth area) coated with 0.2% gelatin solution (Sigma Aldrich, San Luis, MO, USA) were maintained in a 5% CO_2_ incubator at 37 °C until they reached confluence. Then, the cells were treated with 10% of pooled sera from each immunized mice group and incubated at 37 °C (5% CO_2_). Transendothelial electrical resistance (TEER) was evaluated at 2, 6, and 24 h after treatment using the EVOM2 voltmeter with a STX100c electrode (World Precision Instruments, Sarasota, FL, USA). The TEER values in ohms (Ω) of the negative controls (inserts without cells) were subtracted from the values obtained in each insert with cells. Finally, the values were multiplied by the area of the inserts, resulting in the TEER reported in Ω × cm ^2^.

### 2.10. Statistical Analyses

Statistical analyses were performed with Prism 6 software (GraphPad Software Inc, LA Jolla, CA, USA). One-way ANOVA was applied with Bonferroni’s post hoc test to compare results involving several (≥3) groups. Two-way ANOVA followed by Bonferroni’s correction was used when the data involved several groups and more than one variable (time points or concentrations). Differences were considered significant when the *p*-value (*p*) was ≤0.05.

## 3. Results

### 3.1. Intradermal Delivery of the NS1-Based DC-Targeting mAbs

**Figure 1:**
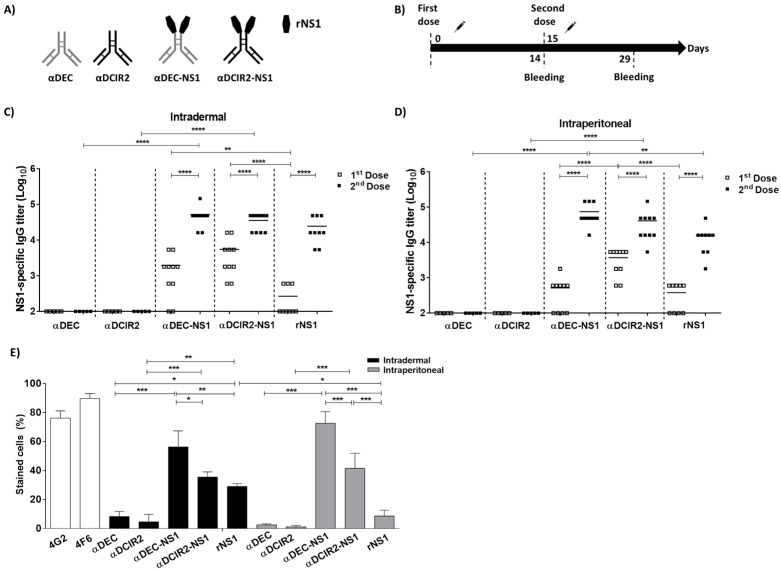
Nonstructural protein 1 (NS1)-specific serum IgG responses in the mice subjected to the tested vaccine regimens. (**A**) Schematic representation of chimeric monoclonal antibodies (mAbs) and recombinant form of type 2 dengue virus NS1 protein (rNS1). (**B**) Schematic representation of the tested vaccine regimen. BALB/c mice were immunized via the intradermal (i.d.) or intraperitoneal (i.p.) route with two doses of the vaccine formulations. Serum samples were collected on the indicated days. (**C** and **D**) NS1-specific serum IgG titers measured by ELISA following i.d. (**C**) or i.p. (**D**) administration. Values represent the individual results (Square symbols) and means (black lines) of the determined IgG titers in log_10_ scale. (**E**) Binding of serum antibodies to native NS1 expressed on the DENV2-infected Vero cells. Diluted serum samples collected 14 days after the last dose were incubated with DENV2-infected cells and subsequently analyzed by flow cytometry. The 4G2 and 4F6 mAbs (specific to E and NS1 proteins, respectively) were used as controls. Statistical significance was determined by one- or two-way ANOVA with Bonferroni’s post hoc test (* *p* < 0.05, ** *p* < 0.01, *** *p* < 0.001, **** *p* < 0.0001).

The successful production (Figure S1A–C) and specific targeting (Figure S1D–E) of αDEC-NS1 and αDCIR2-NS1 to their respective receptors expressed in Chinese hamster ovary (CHO) cells were confirmed under in vitro conditions. We measured the NS1-specific serum antibody responses in BALB/c mice immunized with two doses of the recombinant mAbs fused with NS1 (αDEC-NS1 or αDCIR2-NS1) or equimolar amounts of a purified recombinant form of DENV2 NS1 protein (rNS1) (Figure 1A,B), all combined with poly (I:C) as a stimulus for DC maturation [47]. As controls, mouse groups were also immunized with unfused mAbs (αDEC or αDCIR2). As shown in Figure 1C,D, high titers (>4.5 log) of NS1-specific serum IgG responses were detected in all mouse groups immunized through i.d. and i.p. delivery, especially after the administration of the second dose. In addition, higher anti-NS1 IgG titers were observed after the second dose in mice immunized i.p. with αDEC-NS1 mAb when compared to mice immunized with rNS1 plus poly (I:C). A comparison after the administration of the first vaccine dose showed significant differences between αDCIR2-NS1 and rNS1 groups immunized i.d., and among αDCIR2-NS1 and αDEC-NS1 or rNS1 in the i.p immunized group. We did not observe any statistically significant differences when we compared specific titers in mice immunized by the i.d. or i.p. routes. As expected, unfused mAbs did not induce NS1-specific IgG antibodies.

We next evaluated the binding of NS1-specific antibodies to native NS1 expressed in DENV2-infected Vero cells (Figure 1E). Notably, anti-NS1 antibodies raised in the mice immunized with αDEC-NS1, via either the i.d. or i.p. route, displayed higher binding to the DENV2 NS1 expressed on the surface of infected cells compared to the anti-NS1 serum samples collected from αDCIR2-NS1 or rNS1 immunized groups (Figure 1E). In addition, when compared to i.p., i.d. immunization was more efficient to elicit antibodies to the native protein in the rNS1 immunized group. Taken together, these results indicate that although anti-NS1 antibodies raised after the second dose in all groups are able to recognize the rNS1 by ELISA, there are important differences in their recognition patterns when the native NS1 is expressed by DENV2 infected cells. More relevantly, our findings demonstrated that the i.d. administration of DC-targeting chimeric mAbs induced a similar anti-NS1 response when compared to the one induced by i.p. delivery.

### 3.2. Epitope Specificity, IgG Subclass, and Antigen Avidity of Anti-NS1 Antibodies Raised in the Immunized Mice

**Figure 2:**
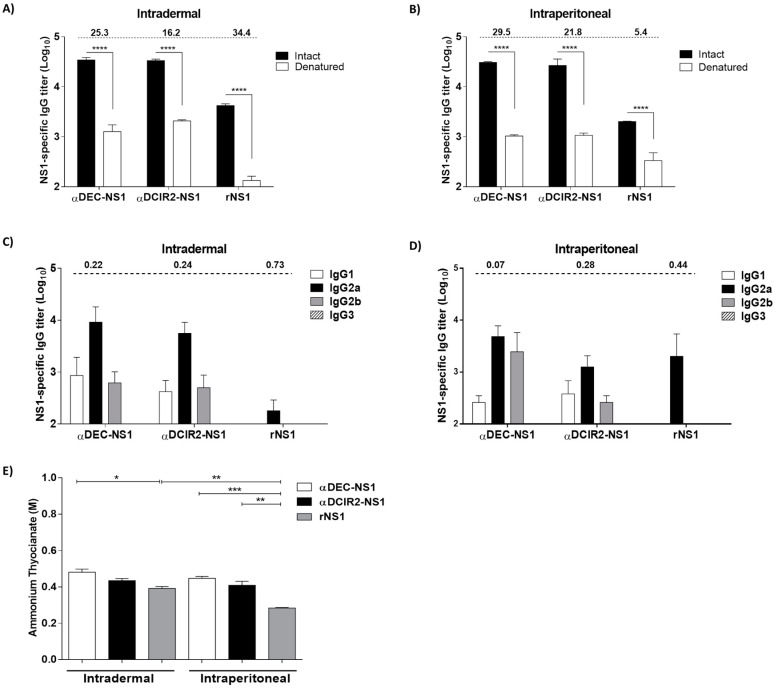
NS1-specific serum IgG antibody responses in the vaccinated mice. Serum antibodies collected from mice immunized via the i.d. (**A**) or i.p. (**B**) routes were evaluated by ELISA using intact (black bars) or heat-denatured (white bars) DENV2 rNS1 protein. The intact/denatured ratios of the IgG titers of each immunization group are indicated on the top of the figure. (**C**,**D**) IgG subclass response in the vaccinated mice. NS1-specific serum IgG1, IgG2a, IgG2b, and IgG3 responses were detected in the mice immunized with αDEC-NS1, αDCIR2-NS1, or rNS1 via i.d. (**A**) or i.p. (**B**) routes. Serum samples were collected 14 days after the second vaccine dose. Values are expressed as the means ± SD of IgG titers (log_10_) (*n* = 5/group). The IgG1/IgG2a ratios of each immunization group are indicated at the top of the figures. (**E**) Antigen avidity for the NS1-specific antibodies raised in vaccinated mice. Values are expressed as the mean ± SD of the concentration of ammonium thiocyanate required to dissociate 50% of bound anti-NS1 antibodies. Serum samples were collected 14 days after the second immunization dose. Significance was determined by one-way ANOVA with Bonferroni’s post hoc test (* *p* < 0.05, ** *p* < 0.01, *** *p* < 0.001, **** *p* < 0.0001).

Antibodies raised in mice immunized i.d. or i.p. with αDEC-NS1, αDCIR2-NS1, or rNS1 preferentially bound to conformational epitopes of DENV2 NS1, as the specific antibody titers were significantly reduced when the NS1 protein was heat-denatured (Figure 2A,B).

Analyses of the NS1-specific serum IgG subclass responses indicated that mice immunized with the chimeric antibodies mounted IgG1, IgG2a, and IgG2b responses (Figure 2C,D). We did not detect the IgG3 subclass in any group. Our results showed that NS1 targeting to the DEC205 or DCIR2 DC subtypes was able to induce efficient class switching, while the administration of rNS1 was only able to induce IgG2a. In order to more precisely compare the profile of the humoral response, we calculated the IgG1:IgG2a ratio (Figure 2C,D). Our results showed that the IgG1:IgG2a ratios were below 1 for all groups, indicating a prevalence of IgG2a antibodies and a more type 1 T helper (Th1) prone immune response.

Next, we measured the avidity of the specific antibodies using different concentrations of ammonium thiocyanate (8 to 0.125 M, Figure S2A). When the concentrations of ammonium thiocyanate required to dissociate 50% of the NS1-bound antibodies were determined (Figure 2E), we observed that the antigen avidity of anti-NS1 antibodies raised in mice immunized with αDEC-NS1 did not differ when i.d. and i.p. immunization routes were compared. On the other hand, anti-NS1 antibodies raised in mice immunized with αDEC-NS1 bound with more avidity to NS1 than the antibodies raised in mice immunized with rNS1, after either i.d. or i.p. delivery. When the anti-NS1 antibodies avidity was compared in the αDCIR2-NS1 immunized groups, a significant difference was observed with the rNS1 group only after i.p. immunization (Figure 2E). Additionally, i.d. vaccination was capable of increasing the avidity of the anti-NS1 antibodies even in the absence of DC targeting (Figure 2E). To confirm these results, we also calculated the areas under the curves (AUC) generated with the all different ammonium thiocyanate concentrations tested (Figure S2B). When the total AUC was considered, we did not observe the difference among the i.d. immunized groups, nor the difference observed between i.p. immunized αDCIR2-NS1 versus rNS1 groups. The results indicate that i.p. immunization with αDEC-NS1, but not by i.d. delivery, induces anti-NS1 antibodies with more avidity than those ones induced by rNS1 immunization. More importantly, we confirmed that i.d. immunization was able to improve antibody avidity in the absence of DC-targeting, as evidenced in the mice i.d. immunized with rNS1 when compared to those immunized by the i.p route (Figure S2B).

Taken together, our results show that immunization with the chimeric αDEC-NS1 or αDCIR2-NS1 mAbs, via either i.d. or i.p. routes, induces anti-NS1 antibodies that recognize conformational epitopes and efficient antibody class switch. In addition, i.d. immunization with rNS1 is able to induce anti-NS1 antibodies with higher avidity when compared to i.p. immunization.

### 3.3. Longevity and Memory Antibody Responses in the Mice Immunized with Chimeric DC-Targeting mAbs

**Figure 3:**
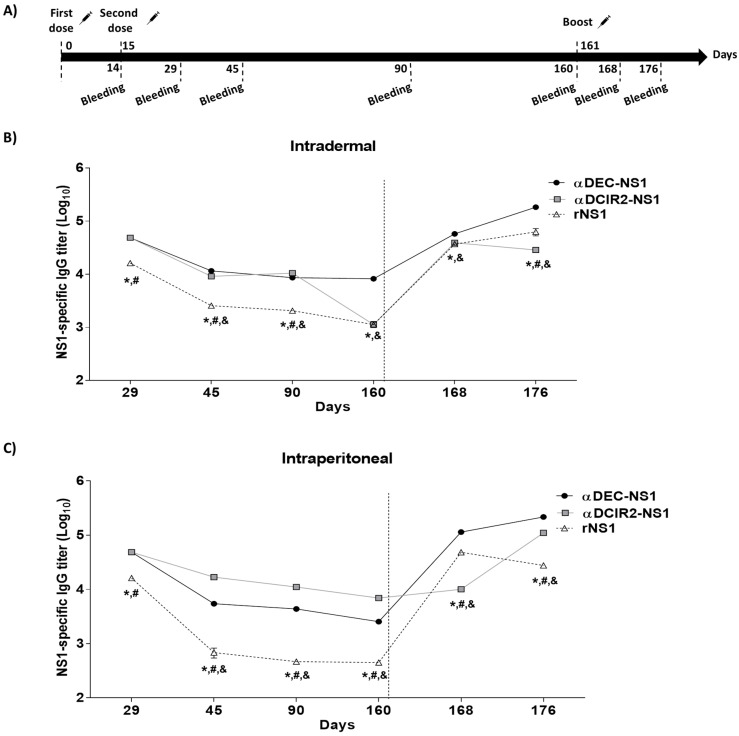
Longevity and boost antibody responses in mice immunized via i.d. and i.p. routes. (**A**) Schematic representation of a prolonged vaccination schedule. (**B**,**C**) NS1-specific IgG titers ± SEM were measured in the pooled serum samples (5 animals/group) collected up to 176 days after the first dose following immunization via the i.d. (**B**) or i.p. (**C**) routes. On day 161, mice were boosted with 1 µg of rNS1 using the same administration route. Significance was determined by two-way ANOVA with Bonferroni’s post hoc test (* *p* < 0.05 comparing rNS1 and αDEC-NS1 groups; # *p* < 0.05 comparing rNS1 and αDCIR2-NS1 groups; & *p* < 0.05 comparing αDEC-NS1 and αDCIR2-NS1 groups (see Figure S3 for comparisons between the i.d. and i.p. routes).

We monitored the longevity and recall responses of anti-NS1 serum IgG in mice vaccinated with αDEC-NS1 or αDCIR2-NS1 after i.d. or i.p. administration (Figure 3A). After the administration of the second dose on day 14, the anti-NS1 IgG titers were monitored from days 45 to 160, and titers greater than 10^3^ were observed among the mice immunized via the i.d. route (Figure 3B). Our results show that specific antibody levels in the αDEC-NS1 and αDCIR2-NS1 groups were reduced between days 29 and 45, and then, they stabilized until day 90. Surprisingly, the anti-NS1 antibodies levels induced in the αDCIR2-NS1 group dropped more than one log on day 160 compared to the αDEC-NS1 group. In the rNS1 immunized group, the anti-NS1 antibody levels were lower than the ones found in the chimeric mAbs immunized groups and significantly decreased over time (Figure 3B). Different results were obtained when the i.p route was analyzed (Figure 3C). In this case, we observed a consistent decrease in anti-NS1 antibody titers in mice immunized with either αDEC-NS1 or αDCIR2-NS1 from days 29 to 160. However, contrary to what was observed in the i.d. immunized group, i.p. immunization led to a more pronounced decrease in specific titers in the αDEC-NS1 when compared to the αDCIR2-NS1 immunized group. A more pronounced reduction was observed in the rNS1 group (Figure 3C). In an attempt to evaluate which immunization route is more efficient in maintaining anti-NS1 titers, we compared the i.d. and i.p. routes for each immunization group (Figure S3). Interestingly, i.d. immunization induced significantly higher anti-NS1 titers than i.p. immunization on days 45 to 160 (Figure S3A). A different pattern was observed in the αDCIR2-NS1 group. In this case, the i.p. route induced significantly higher antibody titers on days 45 and 160, indicating that for this particular mAb, the i.p. route seems more efficient to induce antibodies that circulate for longer periods (Figure S3B). When both routes were compared for the rNS1 immunized groups, again, the i.d route was superior from days 45 to 160 (Figure S3C).

The expansion of memory antibody responses was also measured in the vaccinated mice. Animals were immunized with 1 µg of rNS1 (boost) on day 161 (145 days after the second vaccine dose) using the same administration route (Figure 3A). Figure 3B,C shows that anti-NS1 serum IgG titers increased quickly in all groups, except on the αDCIR2-NS1 i.p. immunized group, as measured one week after the boost dose was administered (day 168) (Figure 3B,C). The highest anti-NS1 antibody titers were observed in the αDEC-NS1 immunized animals, despite the immunization route. A comparison between i.d. and i.p. routes after boost showed that i.p. immunization induced higher anti-NS1 titers in the αDEC-NS1 group on days 168 and 176 (Figure S3A). Figure S3B shows significant differences in the αDCIR2-NS1 group, with the i.d. route performing better on day 168 and i.p. route performing better on day 176. A similar result was observed in the rNS1 immunized group, but in this case, the i.p. route performed better on day 168 and the i.d. route performed better on day 176 (Figure S3C). Altogether, these results indicate that NS1 targeting, especially to the DEC205+ DCs, improves longevity and enhances boost responses using both i.p. and i.d. administration routes.

### 3.4. Safety of αDEC-NS1 and αDCIR2-NS1 Immunization via the i.d. and i.p. Delivery Routes

Immunization via either the i.d. or i.p. routes with chimeric DC-targeting mAbs was monitored with regard to cross-reactivity with platelets, endothelial cell damage, and disturbance of the coagulation pathway. First, we analyzed hematological parameters of the mice subjected to both immunization regimens one week after the last vaccine dose was administered. As shown in Table S1, no significant differences were observed in the total number of leukocytes, granulocytes, or lymphocytes, nor in the hematocrit levels among the mice immunized with chimeric mAbs when compared to the control groups. The numbers of platelets did not differ from those detected in the control mouse groups (Figure S4A,B). Additionally, we measured bleeding and prothrombin times in the mice immunized with chimeric mAbs (Figure S5A,B). Tissue damage following vaccination was evaluated by determining lactate dehydrogenase (LDH) (Figure S5C), glutamic oxaloacetic transaminase (GOT), and glutamic pyruvic transaminase (GPT) (Figure S6A–D) activity levels, and no significant differences were detected among the different immunization groups. Altogether, these results indicate that immunization with NS1-fused chimeric mAbs did not induce significant hematological disorders or tissue damage.

**Figure 4:**
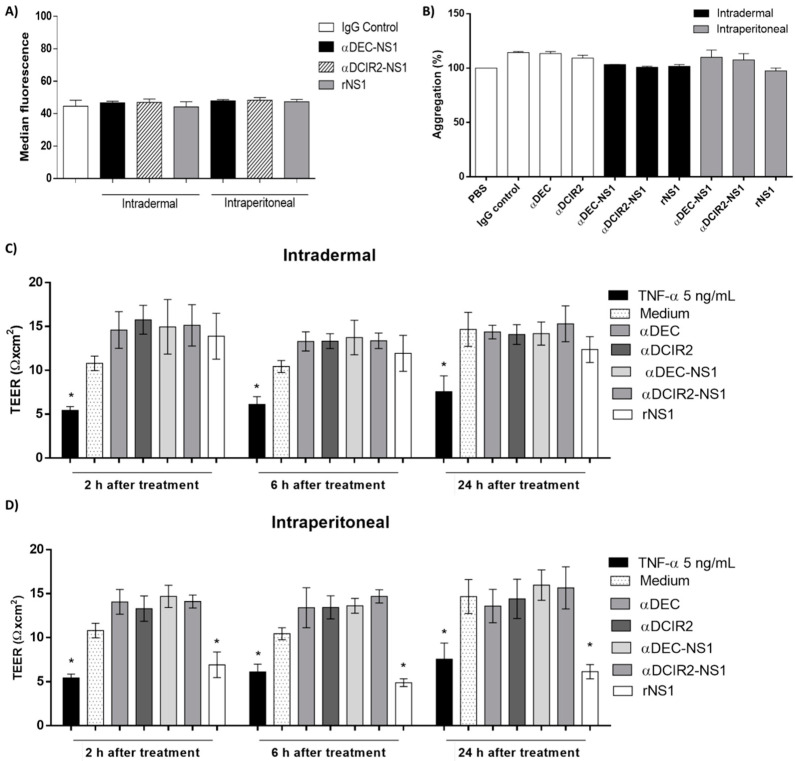
Safety parameters of anti-NS1 immune responses elicited in the mice vaccinated with αDEC-NS1, αDCIR2-NS1, or rNS1 via different immunization routes. (**A**) Binding of anti-NS1 antibodies to platelets in serum samples collected from the mice immunized with αDEC-NS1, αDCIR2-NS1, or rNS1 via the i.d. or i.p. route. The concentration of NS1-specific IgG was adjusted to 10 µg/mL. Platelet binding activity was monitored with an anti-mouse IgG antibody conjugated to Alexa Fluor 488. (**B**) Platelet aggregation inhibition by NS1-specific serum. Plasma enriched with platelets was incubated with anti-NS1 antibodies collected from the vaccinated mice. After incubation, the platelet suspensions were monitored for aggregation following stimulation with 20 μM adenosine diphosphate (ADP). (**C**,**D**) Transendothelial Electrical Resistance (TEER) of human umbilical vein endothelial cells (HUVEC) confluent monolayers incubated with diluted serum samples collected from the mice immunized via i.d. (**C**) or i.p. (**D**) routes. TNFα was used as a positive control. TEER was measured at 2, 6, and 24 h after treatment and values expressed as ohms.cm^2^. Data are showed as the mean ± SEM of two distinct experiments. Significance was determined by one- or two-way ANOVA with Bonferroni’s post hoc test (* *p* < 0.05).

We also evaluated the impact of the induced anti-NS1 responses on platelets and endothelial cell function. As shown in Figure 4A, the in vitro binding of anti-NS1 antibodies to platelets did not lead to significant differences between the serum samples collected from the different immunization groups. Similarly, ADP-induced platelet aggregation promoted in the tested serum samples was not significantly different (Figure 4B and Figure S7). Vascular impairment was measured through an in vitro endothelial barrier model using human umbilical vein endothelial cells (HUVEC) monolayers treated or not with serum samples from the NS1-vaccinated mice. As demonstrated in Figure 4C,D, there was no significant change in the TEER values of the cells treated with serum obtained from mice immunized with αDEC-NS1 or αDCIR2-NS1 regardless of the administration route, at the different time points analyzed. Notably, serum from mice immunized with rNS1 via the i.p. route induced significant TEER reduction at all tested time points, with values comparable to the positive control TNFα (Figure 4D). Altogether, these results indicate that immunization with NS1-based chimeric mAbs did not induce platelet or endothelial dysfunction.

## 4. Discussion

Antigen targeting to DCs using αDEC205 or αDCIR2 mAbs is a strategy reported to increase the immunogenicity of vaccine antigens [3,47,52]. However, these mAbs are usually administered in mice via the i.p. route, which is not adequate for human use. On the other hand, abundant literature confirms the performance of the i.d. immunization route under both preclinical and clinical conditions. The use of the i.d. route is related to the modulation of protective immune responses against several pathogens, including influenza virus [19], *Mycobacterium tuberculosis* [20], *Trypanosoma cruzi* [22], and DENV [21,23,29]. Here, we compared the i.d. and the i.p. delivery routes of αDEC205 and αDCIR2 mAbs on the DC-targeting of the DENV2 NS1 antigen using poly (I:C) as adjuvant. Immunization regimens based on the i.d. and i.p. administration routes showed similar performances. Altogether, our results indicate that the combination of the DC antigen-targeting approach and the i.d. delivery route improves the immunogenicity and safety of anti-DENV subunit vaccine formulations.

To better understand the influence of the i.d. route on antigen targeting to DCs, we used the DENV2 NS1 protein fused to αDEC205 and αDCIR2 mAbs. This strategy based on i.p. delivery was previously reported by our group to lead to increased antigen-specific humoral and cellular immune responses, and induced protective immunity against DENV infection [47]. Here, we focused on the characterization of NS1-specific antibody responses raised in mice immunized i.d. versus i.p. This choice was based on three important characteristics of anti-DENV NS1 antibodies: (i) binding capacity to NS1 expressed on infected cells, which can contribute to the clearance of these cells by the complement system (CS) activation or antibody-dependent cellular cytotoxicity (ADCC); (ii) protective effects against NS1-mediated endothelial cells (EC) dysfunction, and (iii) risks of binding to platelets, ECs, and coagulation factors, which can contribute to dengue pathogenesis [42,53]. Thus, when the magnitude of the antibody responses in mice was assessed, we observed that the i.d. delivery of DENV2 NS1 protein, in isolation or when fused to αDEC205 or αDCIR2 mAbs, induced similar anti-NS1 IgG titers when compared to the titers observed after i.p. immunization. These results are in accordance with those previously described after i.p. vaccination [47] and indicate similar immunogenicity of the NS1 protein when administered by these pathways.

Intradermal immunization has been shown to modulate both antigen avidity and IgG subclass responses [28,54,55]. Under our testing conditions, the i.d. administration of rNS1 plus poly (I:C) increased the avidity of antibodies compared to the antibodies elicited in the i.p. immunized mice. Nonetheless, immunization with the αDEC-NS1 mAb resulted in increased antigen avidity of the anti-NS1 antibodies when the i.p. route was used. Regarding the serum IgG subclass responses, mice immunized via either i.d. or i.p. routes elicited different serum IgG subclasses (IgG1, IgG2a and IgG2b) but showed IgG1:IgG2a ratios smaller than 1, suggesting a predominance of IgG2a and activation of a type 1 T helper (Th1) cells response. Interestingly, murine IgG2a and IgG2b are the most prevalent subclasses induced after virus infection in mice, and they are able to fix complement and induce high FcγR-mediated activity, such as ADCC, which have been related to protection against virus infection. On the other hand, IgG1 does not fix complement in mice and is related to type 2 T helper (Th2) cell response activation [56,57,58,59,60]. Our observations are in agreement with those of previous studies in which i.d. immunization promoted an increase in dermal DC migration to draining lymph nodes (LNs) and the activation of Interferon (IFN)γ- and Interleukin (IL)-2-producing CD4+ T lymphocytes [22,54,55,61]. Nonetheless, it is important to mention that the use of poly (I:C) as the DC maturation stimulus for both immunization regimens may also play a role in skewing the immune response to a more Th1-prone [7,13,47,62]. In fact, our group has already showed that the choice of the adjuvant is very important to modulate the induction of antibody class switch when chimeric mAbs are used to target antigens to DCs [8]. Despite that fact, it has become more evident in the literature that the DEC205+ DCs are more prone to induce a Th1 response than the DCIR2+ DCs. On the other hand, DCIR2+ DCs are specialized in the induction of Tfh cells [63,64].

In flavivirus-infected cells, the NS1 dimer may be associated with the plasma membrane form (mNS1), and thus, it is accessible to circulating anti-NS1 antibodies [43]. Anti-NS1 antibodies binding to mNS1 have been related to virus clearance by the CS or ADCC and to protective immunity against Zika and dengue viruses [60,65,66,67]. In this context, we demonstrated in the present study that immunization with chimeric mAbs induced antibodies that recognized native NS1 expressed on DENV-infected cells. Targeting NS1 to DEC205 enhanced the immunogenicity of the protein and improved the specificity to conformational epitopes found on the native viral protein. Indeed, these data support previous evidence that the genetic fusion of NS1 to mAbs preserved features of the native protein [47]. Altogether, these results indicate that the fusion of DENV NS1 to DC-targeted mAbs improves the quality of the antibody responses elicited in mice immunized via either the i.d. or i.p. routes.

One of the hallmarks of successful vaccines is their ability to induce long-lasting protective immune responses. Previous observations indicated that DCs targeting with αDEC205 mAb induces long-term T helper cell action and modulates memory B cell responses [7]. In the present study, we evaluated whether i.d. administration of NS1 vectorized by αDEC205 and αDCIR2 mAbs had an impact on the longevity of specific serum IgG responses. Monitoring the induction of anti-NS1 IgG titers showed that mice subjected to i.d. immunization with αDEC-NS1 developed higher memory responses than those immunized i.p., as indicated by the induced serum IgG response 5 months after immunization. On the other hand, the response induced by i.d. immunization with αDCIR2-NS1 was similar to the one induced by i.p. immunization for a while, but it dropped by day 160. After boosting the antigen-specific antibody response with an additional load of purified NS1 antigen on day 161 after the first dose (145 days after the second dose), the anti-NS1 specific response was greatly increased and, on day 176 (two weeks after boost), it reached similar or superior levels to those observed on day 29 (two weeks after the second dose). In conclusion, our results demonstrate that the i.d. administration of chimeric αDEC205 mAb confers a similar impact on the longevity of the induced antibody responses as in mice immunized via the i.p. route.

Although several studies have demonstrated vaccine potential for DENV NS1 [39,40,41,42,43], the safety of the vaccine formulation needs to be evaluated under both preclinical and clinical conditions [42]. The DENV NS1 protein has been reported to direct activate ECs promoting vascular hyperpermeability [53,68], to antagonize the C4b complement component as a mechanism of immune evasion [69], and to interact with prothrombin, which can interfere with coagulation cascade and increase the risk of bleeding [68,69,70]. In addition, anti-NS1 antibodies were found to cross-react with platelets, EC, and coagulation factors, which resulted in their depleted numbers or diminished function [42,71,72,73,74]. Under clinical conditions, elevated NS1 levels in dengue-infected patients serum correlate with disease severity [75,76]. Due to its direct or indirect deleterious effects, sNS1 has the potential to induce damage to host tissues and increase the risk of developing severe forms of dengue [42,77]. In this regard, we did not observe any deleterious effects on the NS1-immunized mice using DC-targeting-based formulations. The hematological parameters of the immunized animals, including the number of circulating platelets, hematocrit level, and bleeding and prothrombin times, were similar to those of the control groups. The activity of GOT, GPT, and LDH enzymes, which mainly indicate tissue damage, was unchanged. Additionally, we also compared the effects of these antibodies generated in the i.p. or i.d. immunized mice on platelets and endothelial cells in vitro. No significant cross-reactivity with platelets or alteration to their aggregation capacity in the presence of hyperimmune anti-NS1 serum was found. Interestingly, only serum samples from i.p. rNS1-immunized mice induced TEER reduction, suggesting a potential capacity to alter the endothelial barrier integrity. Taken together, these results indicate that NS1 i.d. vaccination and antigen targeting to DCs tested herein are safe and can reduce or prevent the induction of anti-NS1 antibodies that interfere with platelet and endothelial functions.

Previous reports also confirmed that it is possible to combine a potent NS1-specific humoral response without causing deleterious effects on immunized mice [39,78]. However, in formulations based on the full NS1 protein, it was clear that the adjuvant adopted can influence vaccine safety, and the incorporation of Freund’s adjuvant (FA), which is not approved for use in humans, caused an increase in inflammation parameters and tissue damage in immunized animals [39]. Interestingly, a significant portion of the studies that report anti-NS1 antibodies with cross-reactivity to human proteins also used immunization protocols containing FA as the adjuvant [71,73,78,79]. Thus, the inflammation profile generated during the assembly of the NS1-specific response may influence the antibodies generated against this antigen. Although the threshold between the induction of beneficial versus malefic anti-NS1 antibodies remains an enigma, the choice of the correct adjuvant in vaccine formulations containing NS1 is a key step in the generation of a safe immune response. In this regard, the adjuvant capacity exerted by poly (I:C), combined with NS1 targeting to DCs and i.d. vaccination, proved to be safe.

## 5. Conclusions

In summary, the results demonstrate that i.d. antigen delivery by immunization with αDEC205 and αDCIR2 chimeric mAbs represents an alternative to the i.p. administration without causing a significant decrease in the immunogenicity of the bystander antigen or the induction of deleterious side effects. In particular, DC targeting by fusion of NS1 to αDEC205 followed by i.d. delivery positively impacted the quality of the antigen-specific antibody response, leading to antigen avidity and longevity of the immune response without any indication that would prompt safety concerns. In addition, this work highlights the vaccine potential of DC-targeting mAbs under experimental conditions that may more easily translate to clinical testing and opens opportunities for the development of efficient and safe NS1-based anti-flavivirus subunit vaccines.

## Supplementary Materials

The following are available online at https://www.mdpi.com/2076-393X/8/4/565/s1, Figure S1: Purification and cell-binding activity of the αDEC-NS1 and αDCIR2-NS1 chimeric mAbs. Figure S2: Antigen avidity of the NS1-specific antibodies raised in the vaccinated mice. Figure S3: Statistical comparison of the long-term antibody responses after immunization via the i.d. or i.p. route. Figure S4: Impact of vaccination on the number of circulating platelets in the immunized animals. Figure S5: Bleeding time, prothrombin time, and LDH activity in the vaccinated mice. Figure S6: Hepatic transaminase activity in the serum samples from the vaccinated mice. Figure S7: Interference of platelet aggregation by anti-NS1 antibodies. Table S1: Hematological analysis of the blood samples from the vaccinated mice.
