# Supplementary material for: Intradermal Delivery of Dendritic Cell-Targeting Chimeric mAbs Genetically Fused to Type 2 Dengue Virus Nonstructural Protein 1
Source: Vaccines (Basel). 2020 Oct 1;8(4):565. doi: 10.3390/vaccines8040565 (PMC7712967; doi:10.3390/vaccines8040565)
Supplement: Supplementary file 1 [file vaccines-08-00565-s001.pdf]

SUPPLEMENTARY MATERIAL

1. Methods

1.1. Western Blotting

Protein samples (1 μg) were subjected to polyacrylamide gel electrophoresis (SDS-PAGE, 15%) and transferred to nitrocellulose membranes (GE Healthcare Life Sciences, Carlsbad, USA). Membrane blockade was performed with a PBS-0.05% Tween solution consisting of 5% non-fat milk incubated for 2 h at room temperature. Primary labeling was performed by incubation (1 h) with monoclonal antibody (4F6) that specifically recognizes the DENV NS1 protein [50] or goat anti-mouse IgG conjugated to an enzyme peroxidase (Sigma Aldrich, San Luis, MO, USA) at a 1:5000 and 1:3000 dilution, respectively, using blocking buffer. HRP-conjugated protein A (BioLegend, San Diego, USA) (1:1000 dilution) was used as a secondary marker. Reactive protein bands were identified by exposure of the membranes to a luminol-hydrogen peroxide solution according to the manufacturer's instructions (Sigma Aldrich, San Luis, MO, USA). Images were captured and analyzed with Image Lab™ software (Bio-Rad, Hercules, USA).

1.2. Binding of the Recombinant Mabs to DEC205 and DCIR2 Receptors

The integrity of the control (αDEC and αDCIR2) and chimeric (αDEC-NS1 and αDCIR2-NS1) mAbs was evaluated by binding to the DEC205 and DCIR2 receptors expressed on CHO cells according to previously described methods [47]. Briefly, 1.5×10^4 CHO cells stably expressing DEC205 or DCIR2 receptors were incubated for 45 min with the diluted antibodies at a final concentration of 10, 1 or 0.1 mg/ml on ice. After incubation, the cells were washed twice with PBS supplemented with 2% FBS (Life Technologies) and then incubated for 30 min with conjugated anti-mouse IgG1 antibody conjugated to phycoerythrin (PE) (BD Biosciences) on ice. After two additional washes, 20,000 events were acquired using a FACSCalibur flow cytometer (BD Biosciences, Franklin Lakes, NJ, USA).

1.3. Monitoring tissue damage in the vaccinated mice.

Individual serum samples were used to determine glutamic oxaloacetic transaminase (GOT), glutamic pyruvic transaminase (GPT) and lactate

dehydrogenase (LDH) levels using analytical kits as recommended by the supplier (Bioclin, Belo Horizonte, Brazil).

1.4. Bleeding time and blood cell analyses

For hematological analyses, whole blood samples from the immunized animals were collected (seven days after the second vaccination dose was administered) in tubes containing sodium citrate (7.8%) and used for hematology blood counts with an BC2800vet auto-hematology analyzer (MINDRAY, Shenzhen, China). A portion of each collected blood samples (50 μL) was also used to measure prothrombin time with an analytical Soluplastin kit (Wiener lab, Rosario, Argentina), according to the manufacturer's instructions.

The bleeding time of the immunized animals was determined (4 days after administration of the second vaccine dose) with a 3 mm transection on the tail tip of the animal as previously described [78]. Blood drops were gently collected with filter paper every 30 seconds, and the bleeding time was counted until the recovered blood spots were approximately 0.1 mm in diameter.

2. Figures

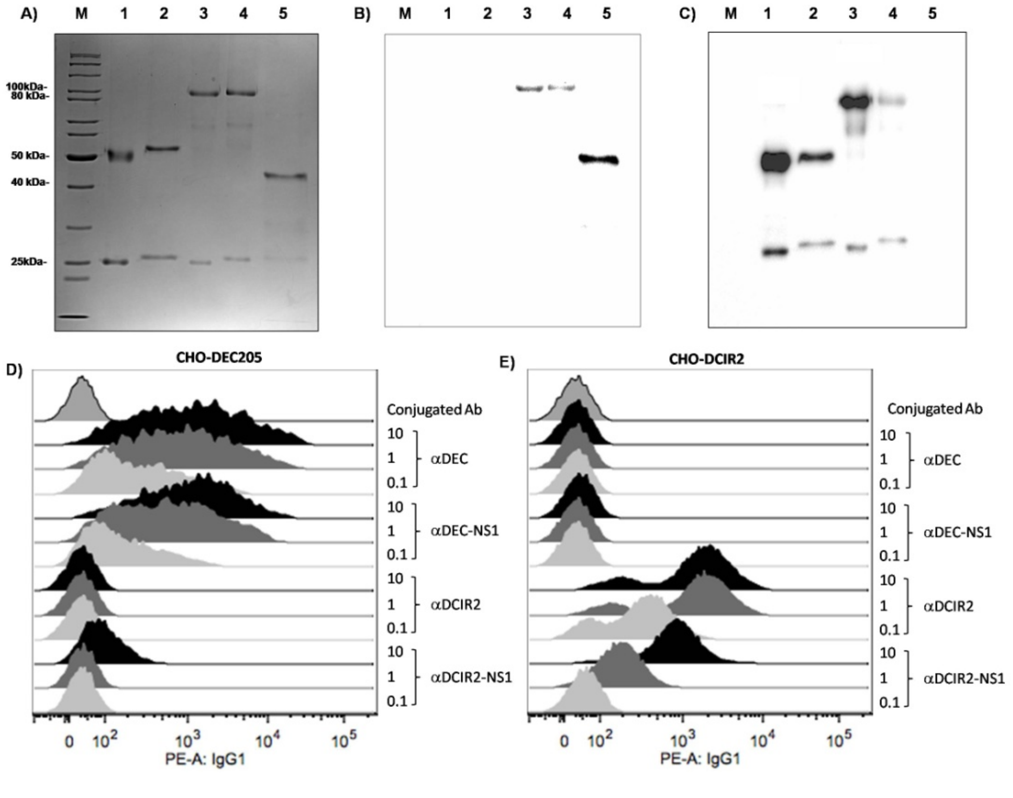

Figure S1. Purification and cell-binding activity of the αDEC-NS1 and αDCIR2-NS1 chimeric mAbs. (**A**) Aliquots (1 μg) of the mAbs or rNS1 were subjected to SDS-PAGE in 15% polyacrylamide gels. (**B**) Immunoblot carried out under denaturing conditions using mAb 4F6 anti-NS1 or (**C**) anti-mouse IgG. (**D** and **E**) The recombinant antibodies were analyzed by flow cytometry (at concentrations of 0.1 μg/ml, 1 μg/ml and 10 μg/ml) for their ability to bind the DEC205 (D) or DCIR2 (E) receptors expressed on CHO cells. Description: M, molecular weight marker; lane 1, αDEC; lane 2, αDCIR2; lane 3, αDEC-NS1; lane 4, αDCIR2-NS1; and lane 5, recombinant NS1.

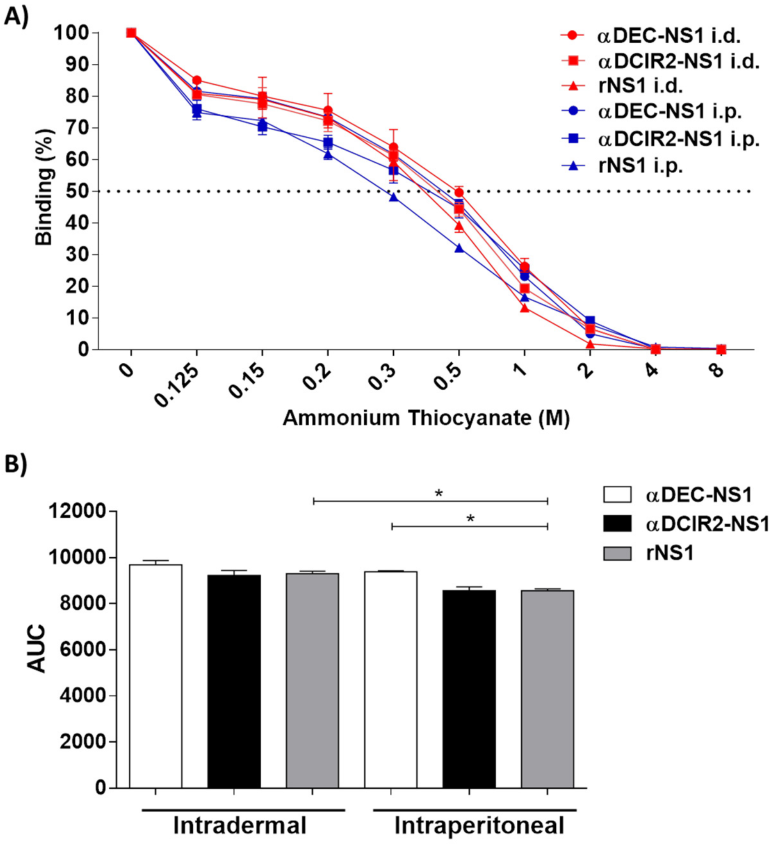

Figure S2. Antigen avidity of NS1-specific antibodies raised in the vaccinated mice. (**A**) Binding profile of NS1-specific antibodies determined by ELISA with a dissociation step using ammonium thiocyanate. Serum samples were collected two weeks after the second immunization dose. Maximum binding (100%) was determined with samples not treated with ammonium thiocyanate. (**B**) Area under curve (AUC) determined with the antibody binding data shown in the figure A. Significance was determined by two-way ANOVA with Bonferroni's post hoc test (* $p < 0.05$).

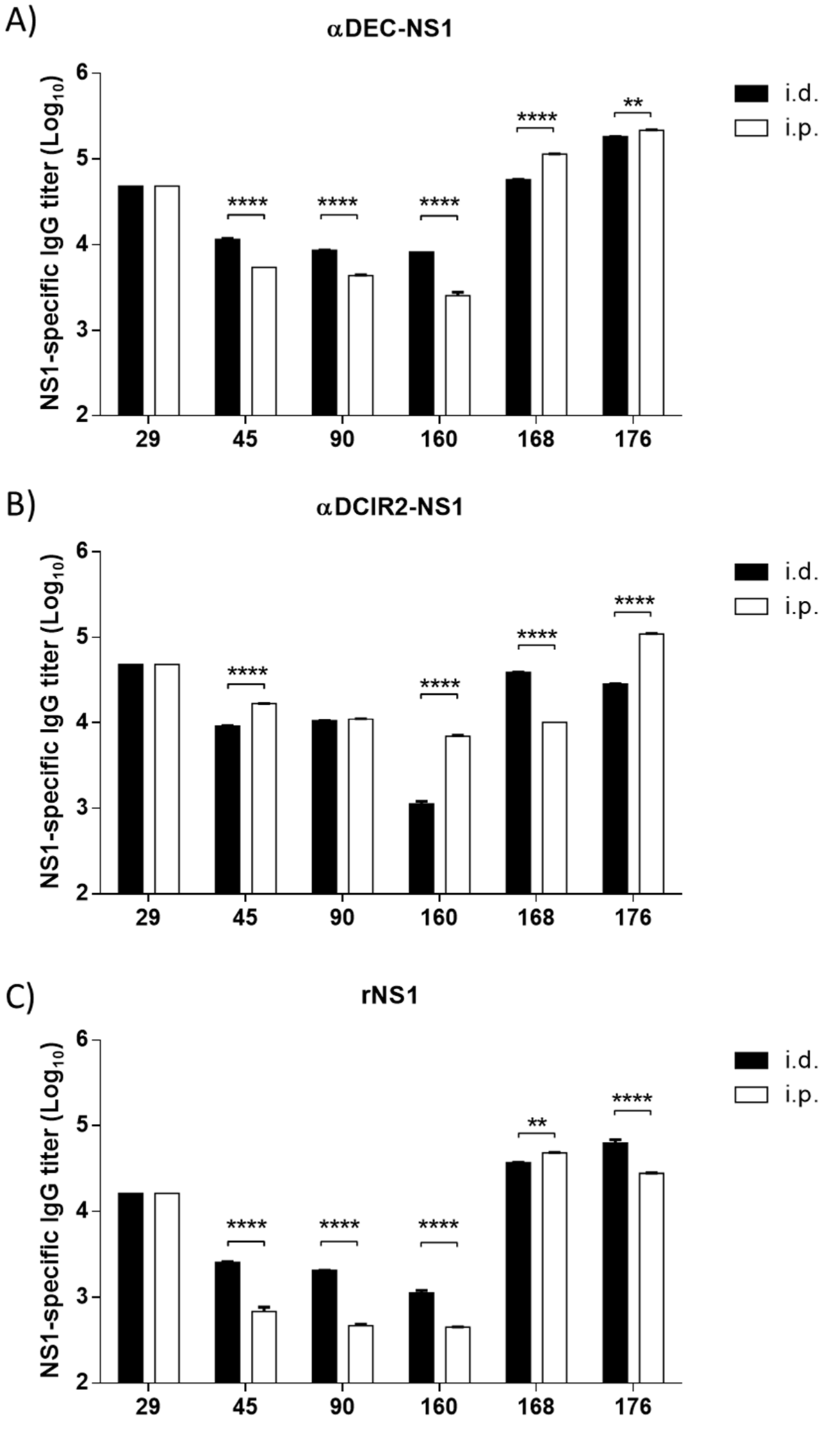

Figure S3. Statistical comparison of the long-term antibody responses after immunization via the i.d. or i.p. routes. IgG NS1-specific titers measured up to day 176 of the immunization regimen in the *α*DEC-NS1 (**A**), *α*DCIR2-NS1 (**B**) and rNS1 (**C**) vaccine groups. Comparisons of the i.d. and i.p. immunization data were performed using two-way ANOVA with Bonferroni's post hoc test (** $p < 0.01$; **** $p < 0.0001$).

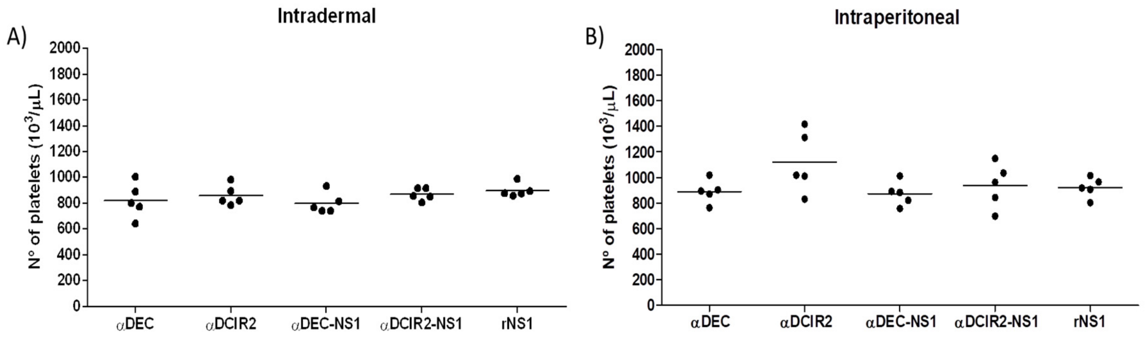

Figure S4. Impact of vaccination on the number of circulating platelets in the immunized animals. Number of platelets in blood samples from the mice immunized via the i.d. **(A)** or i.p. **(B)** route. Samples were collected one week after the second vaccine dose was administered. The number of platelets was determined with a hematological analyzer. The means (bar) and individual (plot) values are expressed as ×10^3 cells/μl of blood. Significance was determined by one-way ANOVA with Bonferroni's post hoc test.

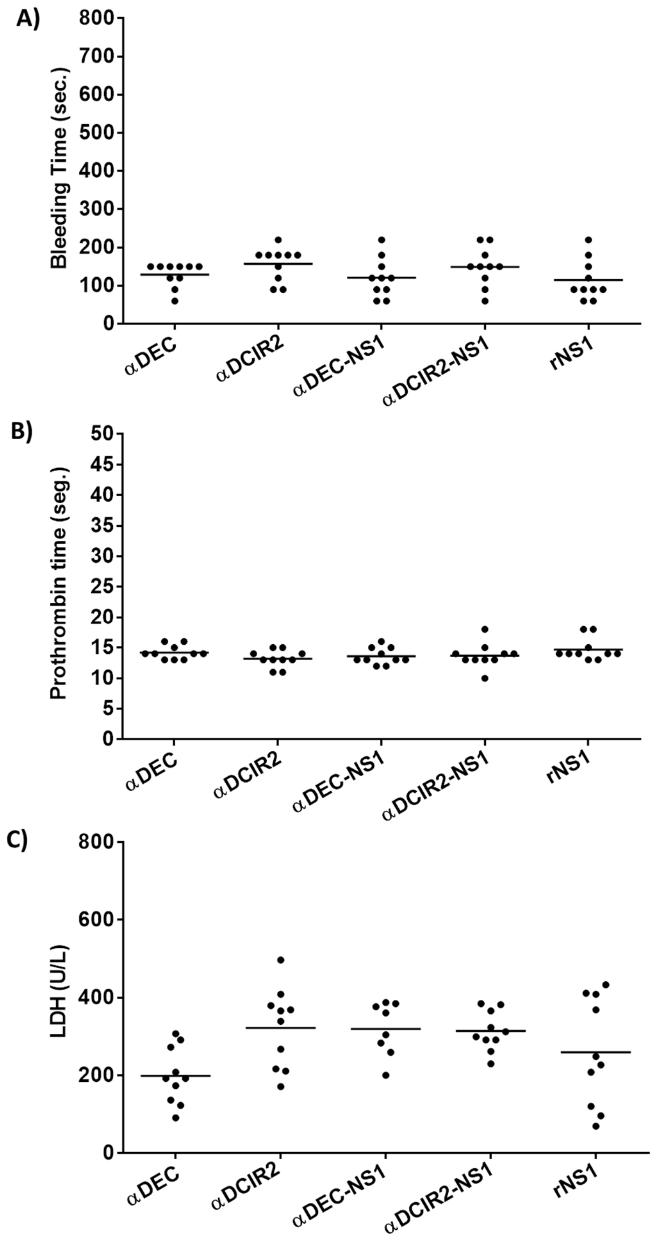

Figure S5. Bleeding time, prothrombin time and LDH activity in the vaccinated mice. All values were measured in the mice immunized with αDEC-NS1, αDCIR2-NS1 or rNS1 via the i.d. route. (**A**) Bleeding times were determined 4 days after administration of the second vaccine dose. Values were determined based on blood collected every 30 seconds from a transection of the mouse tail tip (3 mm). (**B**) Prothrombin times were determined in serum samples collected 7 days after the last vaccine dose was administered. (**C**) Lactate dehydrogenase (LDH) activity was measured in the serum samples collected 7 days after the last vaccine dose was administered. Significance was determined by one-way ANOVA with Bonferroni's post hoc test.

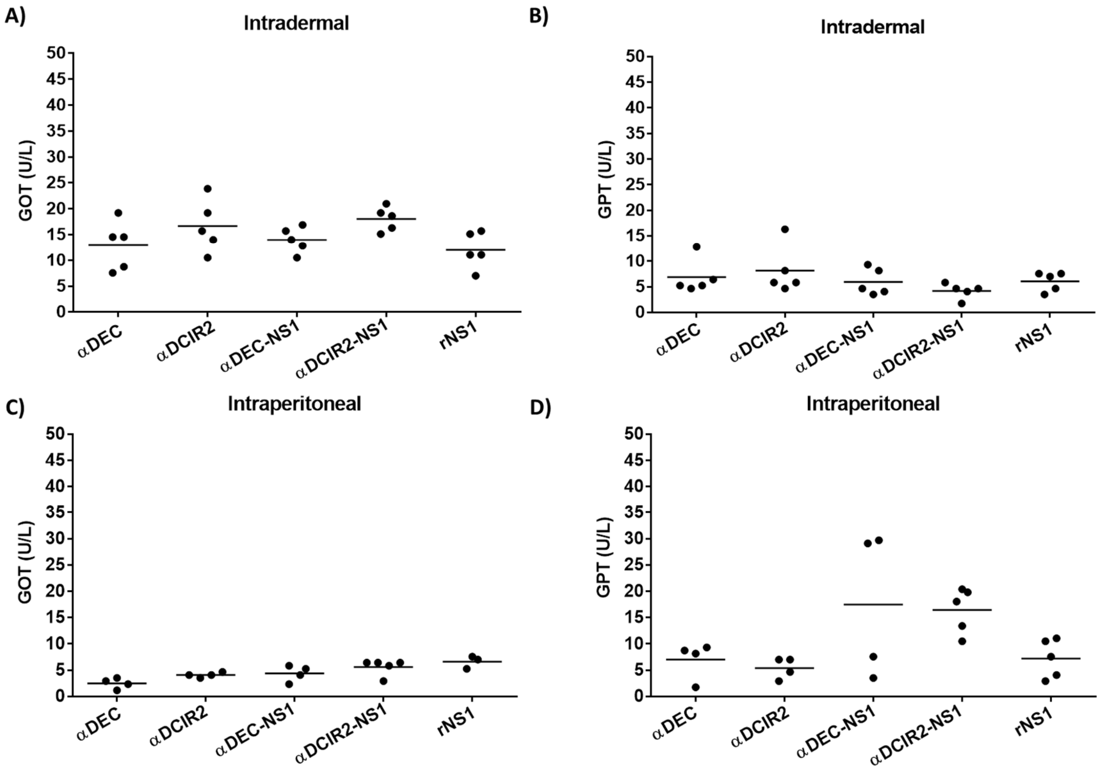

Figure S6. Hepatic transaminase activity in the serum samples from the vaccinated mice. GOT (**A** and **C**) and GPT (**B** and **D**) transaminase activity levels were measured in the combined sera from the immunized mice (n=5) 7 days after the last vaccine dose was administered. The results are expressed as the means (lines) and individual values (plot) of the unit of enzyme per liter of serum (U/L). Significance was determined by one- and two-way ANOVA with Bonferroni's post hoc tests.

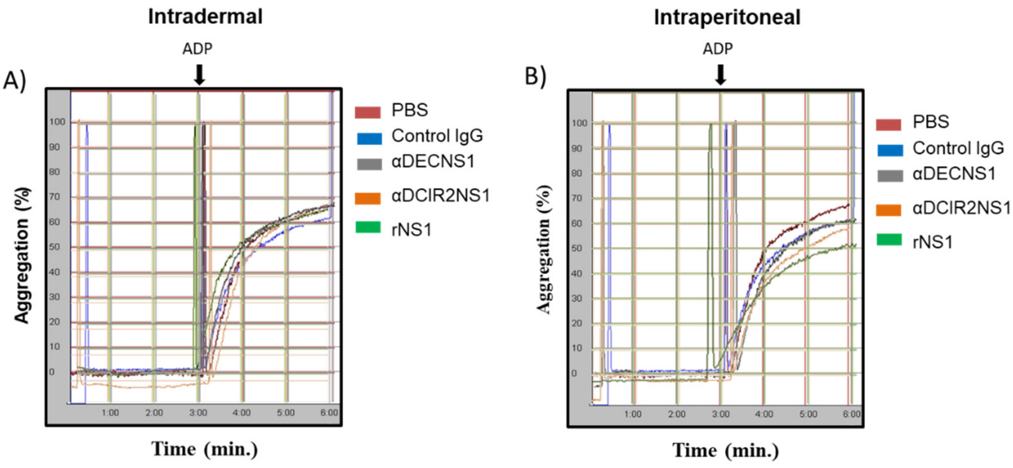

Figure S7. Interference of platelet aggregation by anti-NS1 antibodies. Platelet aggregation was determined by monitoring platelet-rich plasma (PRP) mixed with serum samples (10%) obtained from the mice immunized via i.d.

(**A**) or i.p. (**B**) route. The graphs are representative of each tested serum sample evaluated in an aggregometer. ADP was added to induce platelet aggregation.

Table S1. Hematological analysis of the blood samples from the vaccinated mice. Blood samples from the mice immunized with αDEC-NS1, αDCIR2-NS1 or rNS1 via i.d. or i.p. routes were obtained 7 days after administration of the second vaccine dose. Data are presented as the mean ± SD of individual samples. Description: WBC, white blood cell count; LYM, lymphocytes; MON, monocyte; GRAN, granulocytes; RBC, red blood cells; HBC, hemoglobin; HCT, hematocrit level; PLT, platelet count. Significance was determined by one-way ANOVA with Bonferroni's post hoc test for each hematological parameter.

HEMATOLOGICAL ANALYSES										
Hematological parameters	Intradermal					Intraperitoneal				
	αDEC	αDCIR2	αDEC-NS1	αDCIR2-NS1	rNS1	αDEC	αDCIR2	αDEC-NS1	αDCIR2-NS1	rNS1
WBC	13.68 ± 5.15	9.9 ± 2.31	11.04 ± 2.94	10.38 ± 4.35	9.7 ± 0.8	6.42 ± 0.41	9.1 ± 3.0	6.44 ± 1.32	6.68 ± 1.40	11.32 ± 4.28
LYM	10.48 ± 3.9	7.8 ± 1.67	8.54 ± 2.26	8.64 ± 3.42	7.16 ± 0.77	4.72 ± 0.93	7.14 ± 2.16	5.14 ± 1.17	4.96 ± 0.95	8.38 ± 3.40
MON	0.46 ± 0.2	0.32 ± 0.13	0.34 ± 0.05	0.24 ± 0.15	0.32 ± 0.04	0.28 ± 0.24	0.28 ± 0.14	0.14 ± 0.05	0.24 ± 0.11	0.48 ± 0.47
GRAN	2.76 ± 1.1	1.78±0.56	2.16 ± 0.69	1.5 ± 0.88	2.22 ± 0.40	1.42 ± 0.77	1.68 ± 0.80	1.16 ± 0.19	1.48 ± 0.42	2.46 ± 1.40
RBC	9.66 ± 0.77	9.39±0.61	10.1 ± 0.34	9.56 ± 3.07	9.56 ± 0.26	10.30 ± 0.88	10.05 ± 0.83	9.64 ± 0.56	9.92 ± 0.67	9.84 ± 0.88
HGB	15.48 ± 1.10	15.26± 1.17	15.8 ± 0.40	15.02 ± 4.92	14.64 ± 0.40	16.36 ± 1.34	15.6 ± 1.4	14.82 ± 0.85	15.54 ± 1.14	15.06 ± 1.65
HCT	46.08± 3.35	46.26±4.11	48.1 ± 1.31	46.02 ± 14.76	44.98 ± 1.0	50.42 ± 4.4	48.02 ± 4.0	46.5 ± 3.12	48.64 ± 3.15	46.6 ± 4.86
PLT	822.6 ± 135.21	861 ± 79.83	800 ± 80.62	869.8 ± 267.3	898.6 ± 51.9	889.4 ± 90.95	1117.2 ± 240.33	872.4 ± 94.34	936.6 ± 173.88	921 ± 78.70
